# Supplementary material for: A self-supervised multimodal deep learning approach to differentiate post-radiotherapy progression from pseudoprogression in glioblastoma
Source: Sci Rep. 2025 May 17;15:17133. doi: 10.1038/s41598-025-02026-7 (PMC12085623; doi:10.1038/s41598-025-02026-7)
Supplement: Supplementary file 1 — Supplementary Information. [file 41598_2025_2026_MOESM1_ESM.docx]

# Supplementary Methods

## Self-supervised Training

The self-supervised pre-training process is formulated as a multi-objective loss function wherein the ViT-based encoder learns two proxy tasks, namely contrastive learning and context restoration. The selection of a transformer-based encoder instead of its conventional CNN-based counterpart is attributed to its capability of capturing global context and long-range dependencies in high-dimensional inputs, owing to its attention mechanism. During pre-training, the multimodal MR volumes (T2-FLAIR and T1CE) for each subject are stacked in the channel dimension. Subsequently, the volumes are augmented twice with random patch swapping and adding random Gaussian noise, resulting in two views for each subject. The augmented views are then employed in the proxy tasks: context restoration, and contrastive learning. Context restoration aids the encoder in grasping the structural intricacies and anatomical context of different brain regions. In parallel, self-supervised contrastive learning endeavors to learn representations that encapsulate discriminative and pertinent information from the data

The self-supervised pre-training process is designed as a multi-objective loss function, where the ViT-based encoder is tasked with learning two surrogate objectives: contrastive learning and context restoration. A transformer-based encoder is chosen over traditional CNNs due to its attention mechanism, which excels at capturing global context and long-range dependencies in high-dimensional data. During pre-training, multimodal MR volumes, specifically T2-FLAIR and T1CE for each subject, are stacked along the channel dimension. These volumes are then augmented twice by applying random patch swapping and injecting random Gaussian noise, creating two distinct views per subject. These augmented views are subsequently used in the two proxy tasks, namely context restoration, and contrastive learning.

**Context Restoration**: The context restoration task functions as a robust pretext task, leveraging a decoder module composed of transposed convolution layers to reconstruct the encoded augmented volumes. This process facilitates the encoder's comprehension of the structural and anatomical context of brain regions. The reconstruction is optimized by minimizing the L1 loss, which quantifies the difference between the reconstructed volume and the original input ground truth, thereby refining the encoder's ability to capture detailed and accurate representations.

**Contrastive Learning**: The primary objective of self-supervised contrastive learning is to derive representations that encapsulate significant and practical information about the data. This is achieved using contrastive loss, which ensures that representation vectors from two augmented views of the same volume are highly similar, while those from different volumes are distinctly dissimilar. To generate the representation vector $z$, a linear projection head is appended to the output of the ViT encoder. The contrastive loss between a pair of representation vectors $z_{i}$ and $z_{j}$ is mathematically defined as:

$$\log\frac{\exp\left( {\text{sim}\left( z_{i},z_{j} \right)}/\tau\right)}{\sum_{k=1}^{2N} \mathbb{I}_{\left[ k\neq i \right]}\mathrm{ex}p \left( {\text{sim}\left( z_{i},z_{k} \right)}/\tau\right)},$$

Where, $N$ represents the batch size, $\tau$ is a temperature parameter controlling the scaling of similarities, sim$\left( x,y \right)$ denotes the cosine similarity function, and $\mathbb{I}_{\left[ k\neq i \right]}$ is an indicator function that outputs 0 when $k = i$ and 1 otherwise. The contrastive loss promotes both intra-class compactness and inter-class separability in the learned representations.

Given the structural similarities of the brain across individuals, the contrastive loss is further regularized by the reconstruction loss. This regularization ensures that the learned representations maintain anatomical coherence while preserving discriminative features, aligning the model's learning objectives with the inherent characteristics of brain anatomy.
